# Factors Associated With Fear of Cancer Recurrence in Family Caregivers of Cancer Survivors: A Systematic Review

**DOI:** 10.3389/fpsyg.2021.625654

**Published:** 2021-07-16

**Authors:** Louise O'Rourke, Peter L. Fisher, Sophie Campbell, Amelia Wright, Mary Gemma Cherry

**Affiliations:** ^1^Department of Primary Care and Mental Health, University of Liverpool, Whelan Building, Quadrangle, Liverpool, United Kingdom; ^2^Clinical Health Psychology Service, Linda McCartney Centre, Liverpool University National Heath Service Foundation Trust, Liverpool, United Kingdom

**Keywords:** cancer survivors, family caregivers, fear, recurrence, systematic review

## Abstract

**Objective:** Fear of cancer recurrence (FCR) is a significant concern for family caregivers of cancer survivors and is associated with many adverse outcomes, including increased emotional distress and poorer quality of life. Although several theoretical models have been proposed to account for FCR in cancer survivors, their applicability to caregivers is unknown. The aim of this review was to identify clinical, demographic and psychological factors that are associated with, and predict, FCR in caregivers of cancer survivors.

**Method:** AMED, CINAHL, Medline, PsycINFO, and Scopus were systematically searched for relevant studies reporting quantitative data on factors associated with FCR or similar constructs (e.g., worry or anxiety about cancer recurrence) in family caregivers of adult cancer survivors. Included studies were assessed for methodological quality using a standardized checklist adapted from the Agency for Healthcare Research and Quality.

**Results:** Sixteen studies, half of which were cross-sectional, were included and summarized narratively. Non-modifiable factors, including age (*n* = 6) and treatment modality (*n* = 4), were found to be associated with increased FCR. Significant positive associations were also reported between illness perceptions and FCR (*n* = 3). However, there was heterogeneity across included studies with regards to factors examined and most were conducted in the USA. There were also several methodological limitations to the included studies.

**Conclusions:** Research examining FCR in caregivers of cancer survivors has predominantly focused on demographic and clinical factors. Given the paucity of research exploring the psychological mechanisms underpinning FCR, future research should investigate theoretical underpinnings of FCR in caregivers of cancer survivors to support the development of psychological interventions for this population.

**Systematic Review Registration:** PROSPERO, identifier [CRD42019119729].

## Introduction

Although improvements in cancer care have led to earlier diagnosis and more effective, targeted medical treatment (Arnold et al., 2019), family caregivers of survivors continue to experience adverse effects of the illness, both physically and psychologically (Pitceathly and Maguire, 2003; Kurtz et al., 2004; Girgis and Lambert, 2009). Specifically, cancer caregiving responsibilities can result in issues such as pain, fatigue, financial difficulties and social isolation (Girgis and Lambert, 2009; Stenberg et al., 2010). One of the most distressing concerns for survivors and their families is fear of cancer recurrence (FCR) (Simard et al., 2010), defined as “fear, worry, or concern about cancer returning or progressing” (Lebel et al., 2017). Prevalence of FCR is high in family caregivers (Yeo et al., 2004) and can be higher than for the cancer patients (Longacre et al., 2012; Gold et al., 2016). Managing worries about cancer returning is a commonly-reported unmet need for caregivers (Girgis et al., 2013; Turner et al., 2013; Balfe et al., 2016), which is associated with elevated emotional distress (Longacre et al., 2012) and poorer quality of life (QoL) (Simard et al., 2013).

Although psychological interventions for FCR have been widely researched for cancer survivors (Simard et al., 2013; Maheu and Galica, 2018), there is currently little evidence to support the utility of specific psychological interventions for family caregivers experiencing FCR (Simonelli et al., 2017). To develop more effective interventions for this patient group, we must first understand the psychological processes that underpin and maintain FCR in caregivers of cancer survivors. Much of what we know about these processes is derived from research investigating FCR in patients. Many of the theoretical frameworks proposed to account for FCR in cancer survivors consist of similar components, including internal (e.g., physical symptoms, treatment side effects) and external (e.g., clinical follow-up) cues that trigger a cognitive response associated with FCR (Simonelli et al., 2017). Following an appraisal of such cues, a variety of coping responses, some less helpful than others, are implemented which are influenced by the social environment and other contextual factors (Lee-Jones et al., 1997; Simonelli et al., 2017). Such coping responses may include avoidance, limited future planning, symptom checking and misinterpretation of symptoms, and reassurance seeking from health professionals and family members, which in the longer term can increase FCR (Lee-Jones et al., 1997).

Similar to cancer survivors, caregivers often engage in unhelpful coping behaviors such as avoidance of cancer-related discussions, reluctance to make plans for the future and reassurance seeking (Lambert et al., 2013; LeSeure and Chongkham-ang, 2015). Furthermore, although caregivers do not experience internal cancer-related cues (e.g. cancer symptoms or delayed treatment effects), the cancer journey is experienced by the family as a whole (Kayser et al., 2007). Therefore, caregivers are often aware of survivors' physical experiences of cancer diagnosis and treatment, through helping patients to manage physical symptoms such as treatment side effects (LeSeure and Chongkham-ang, 2015). Caregivers are exposed to many external cues and situations which may trigger FCR, including cancer-related conversations, media references to cancer, appointments with health professionals and survivors' follow-up appointments and feeling unwell themselves (Simard and Savard, 2009).

Although many components of the FCR models will be applicable to understanding FCR experienced by caregivers, some may not be relevant and there may be other factors which are only relevant to caregivers of cancer survivors. To date, only two reviews have examined FCR in caregivers (Simard et al., 2013; Maheu and Galica, 2018). Maheu and Galica briefly summarized literature regarding factors associated with FCR in caregivers, but did not take a systematic approach to identify or analyse data. Simard and colleagues conducted a systematic review of quantitative studies examining FCR in adult cancer survivors, within which they briefly summarized the results of nine studies, published prior to 2011. Collectively, the two previous reviews indicate that non-modifiable factors such as caregiver age and gender, and treatment type, may be associated with caregiver FCR. However, a systematic synthesis of contemporaneous studies examining correlates and predictors of caregivers' FCR does not exist. This systematic review aims to address this gap by critically appraising and synthesize the findings of quantitative studies investigating any demographic, clinical and psychosocial correlate or predictor of FCR in adult family caregivers of adult cancer survivors.

## Materials and Methods

### Review Conduct and Reporting

Review conduct and reporting adhered to recommendations by Centre for Reviews Dissemination (2009) and Preferred Reporting Items for Systematic Reviews and Meta-Analyses (PRISMA) guidance (Moher et al., 2009). The protocol was registered on the international prospective register of systematic reviews, Prospero, in January 2019 (reg. number CRD42019119729) and can be accessed at https://www.crd.york.ac.uk/PROSPERO.

### Search Strategy

AMED, CINAHL, Medline, PsycINFO, and Scopus were systematically searched for published literature using the following search terms: partner (partner^*^, couple^*^, spous^*^, dyad^*^, carer, caregiver, care-giver, care giver, caregiv^*^, husban^*^, wife or wives) and (fear^*^ or worr^*^ or anxiet^*^ or concer^*^ or afraid) and (recur^*^ or relaps^*^ or reoccur^*^ or return^*^ or progress^*^) and (cancer^*^ or tumor^*^ or tumor^*^). There were no restrictions placed on publication date. Searches were repeated in March 2020 to identify any new publications relevant to the review question.

### Inclusion and Exclusion Criteria

To be included in the review, studies had to report quantitative data on factors associated with FCR or similar constructs (e.g., worry or anxiety about cancer recurrence) in adult family caregivers (partners, family members, and close friends) of adult cancer survivors (both aged ≥18 years). Patients were classed as cancer survivors if they had received a diagnosis of cancer and had not been diagnosed with a secondary cancer. Articles had to be published in English in a peer-reviewed journal. Studies were excluded if cancer patients had not yet received treatment, in order to ensure findings were deemed to be taken from a survivorship phase. Studies which did not report data separately for cancer survivors were also excluded (e.g., studies reporting data from survivors and patients with metastatic disease). All case studies, commentaries, conference abstracts, dissertations, editorials, qualitative studies, and review articles were excluded.

### Screening and Selection

Two reviewers (LOR and AW) independently assessed the titles and abstracts of potentially relevant papers. The reviewers then independently reviewed the full-text papers against the inclusion and exclusion criteria. Papers which did not meet the inclusion criteria were removed. Discrepancies (*n* = 3) were discussed with the wider research team (MGC, PF, SC) until a negotiated conclusion was reached.

### Data Extraction

For each study, relevant demographic, methodological and summary data were extracted using a standardized data extraction form by LOR and independently checked for accuracy by AW. Uncertainty (*n* = 1) was resolved through discussion with the wider research team. Authors were contacted if data were unclear or had not been reported within the paper. The following information was extracted: (i) author, (ii) year of publication, (iii) study design, (iv) clinical and treatment characteristics of the survivor (diagnosis, stage, time since diagnosis and treatment type), (v) caregiver demographics (age, gender, ethnicity, and relationship length), and (vi) main findings, including correlates and predictors of FCR. Where studies reported multiple analyses, only data from the most complex relevant multivariate analyses were extracted. This is because multivariate analyses that eliminate potential sources of confounding through statistical control of multiple potential covariates are considered stronger tests of association than univariate analyses. Studies that reported data from the same larger database, but focused analyses on different outcomes were interpreted and referred to as separate studies, with their linked status noted. Correlates and predictors were grouped under the following headings: (i) demographic factors (including age, gender and ethnicity); (ii) clinical factors (treatment, cancer stage, co-morbidities and medical follow-up); and (iii) psychosocial factors (emotional distress, interpersonal factors (including FCR in patients), stress and coping, quality of life and psychological beliefs). Data were analyzed narratively; heterogeneity in study findings precluded meta-analysis.

### Risk of Bias

Studies were assessed for risk of bias using a quality appraisal tool adapted from the Agency for Healthcare Research and Quality (Williams et al., 2010), which assesses risk of bias in studies across various domains relevant to research with physical health populations. This tool considers risk of bias across key methodological areas, such as sample selection, size, description, handling of missing data and analysis (Taylor et al., 2015), thus allowing for comparison of studies across domains. Two reviewers (LOR and AW) separately assessed risk of bias in the included studies. Uncertainty (*n* = 4) was resolved through discussion with the wider research team (MGC, PF, SC). In line with Centre for Reviews Dissemination (2009) guidance, studies were not excluded based on outcome of the risk of bias assessment.

## Results

**Figure 1:**
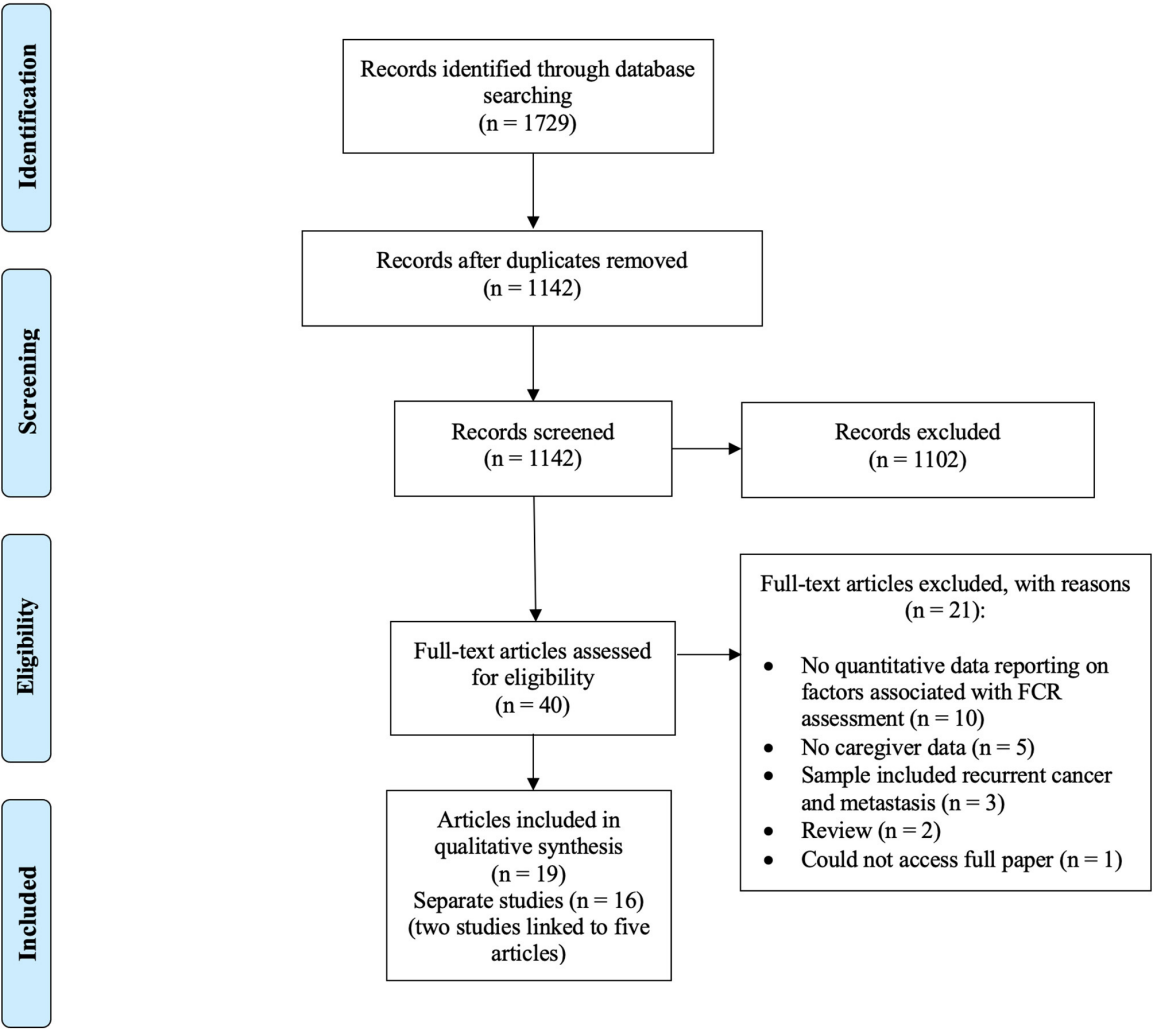
Flowchart of literature search (based on PRISMA guidelines; Moher et al., 2009).

The search strategy identified 1,729 potentially relevant records. After exclusion of duplicates and screening of titles and abstracts, 40 potentially eligible articles remained. After reviewing their full-text, eight articles, reporting seven studies, were identified for inclusion for review. Nine studies were identified during the updated search, resulting in the inclusion of 19 articles, reporting 16 studies^1^. The process of identification of papers to inclusion for review is summarized in Figure 1. Demographic, clinical and psychosocial factors examined by each study are summarized in Table 1.

**Table 1:** Measures of demographic, clinical, and psychosocial factors.

**Correlate or predictor variables**		**Studies analyzing correlate or predictor variables**	**Studies with significant results (*n*)**
***Demographic*** **(*****n*** **=** **12 studies reported in 13 articles)**			
	Age	sig. (29, 32, 34, 39, 42, 43) n.s. (26, 27, 28, 30, 38, 40, 41)	6
	Gender	sig. (42) n.s. (32, 40, 41)	1
	Ethnicity	sig. (28) n.s. (27, 32)	1
	Education	sig. (27) n.s. (26, 28, 32, 38, 41, 43)	1
***Clinical*** **(*****n*** **=** **14)**			
Treatment	Time since diagnosis	sig. (26, 42) n.s. (27, 29, 32, 39, 40, 43)	2
	Treatment type	sig. (26, 28, 35, 42) n.s. (32, 38, 43)	4
	Medical follow-up	sig. (36, 37)	2
Cancer Stage	Cancer stage	n.s. (26, 28, 26, 32, 38-42)	0
	Cancer severity	sig. (29)	1
Comorbidities	Comorbidities	sig. (26, 28) n.s. (32, 39, 40)	2
	Survivor physical health	sig. (29)	1
***Psychosocial*** **(*****n*** **=** **15 studies reported in 18 articles)**			
Emotional distress	Anxiety	sig. (29, 34, 36)	3
	Emotional distress	sig. (41)	1
Interpersonal factors	Survivor/caregiver FCR	sig. (26, 29, 30, 32, 33, 34, 36, 41, 43) n.s. (35, 38)	8 studies reported in 9 articles
	Relationship quality	sig. (34) n.s. (38)	1
	Social support	sig. (26) n.s. (30, 31, 32, 42)	1
	Loneliness	sig. (42)	1
	Communication	sig. (27, 33, 34, 37, 44)	4 studies reported in 5 articles
	Spousal negative affect	sig. (34)	1
Stress and coping	Stress	sig. (30, 31, 32, 42)	2 studies reported in 4 articles
	Coping strategies	sig. (39, 40)	2
Quality of life		sig. (29, 30, 31, 42, 43)	4 studies reported in 5 articles
Psychological beliefs	Meaning of illness	sig. (30, 31, 32)	1 study reported in 3 articles
	Illness perceptions	sig. (39, 40, 44)	3

*sig, significant results; n.s., Non-significant results*.

### Study Characteristics

The main characteristics of the studies are shown in Table 2. Nine studies, reported in 12 articles, were conducted in the USA (Mellon and Northouse, 2001; Mellon et al., 2006, 2007; Kim et al., 2012; Boehmer et al., 2016; Janz et al., 2016; Cohee et al., 2017; Soriano et al., 2018a,b, 2019; Perndorfer et al., 2019; Wu et al., 2019). The remainder were conducted in Taiwan (Chien et al., 2018), UK (Hodges et al., 2009; Dempster et al., 2011; Graham et al., 2016), Ireland (Maguire et al., 2017), The Netherlands (van de Wal et al., 2017) and China (Xu et al., 2019). Studies used a convenience or purposive sampling strategy and were either cross-sectional (Mellon and Northouse, 2001; Mellon et al., 2006, 2007; Dempster et al., 2011; Kim et al., 2012; Boehmer et al., 2016; Janz et al., 2016; Cohee et al., 2017; Maguire et al., 2017; van de Wal et al., 2017; Soriano et al., 2018a) or longitudinal surveys (Hodges et al., 2009; Graham et al., 2016; Chien et al., 2018; Soriano et al., 2018a,b, 2019; Perndorfer et al., 2019; Wu et al., 2019; Xu et al., 2019). Seven studies recruited patients with breast cancer, four with head and neck cancer, three with prostate cancer, and two with mixed cancer diagnoses. The shortest time since diagnosis or treatment was 90 days (Wu et al., 2019), whilst the longest time was 7.3 years (*SD* 3.6) (Boehmer et al., 2016).

**Table 2:** Study characteristics.

			**Caregiver**			**Survivor**			
**Author, (year), country**	**Design**	***n***	**Age** **(years) (SD)**	**Gender,** ***n* (%)**	**Relationship to survivor, *n* (%)**	**Cancer type**	**Stage,** ***n* (%)**	**Time since diagnosis ** ** (SD)**	**Treatment,** ***n* (%)**
Boehmer et al. (2016), USA	Cross-sectional	H^a^ 43 M^b^ 124	*M* = 62.4 (8.0) *M* = 55.8 (9.3)	Female: 7 (16.3) Male: 36 (83.7) Female: 116 (93.5) Male: 8 (6.5)	Partner: 36 (83.7) Child: 3 (7.0) Sibling: 2 (4.7) Parent: 1 (2.3) Friend: 1 (2.3) Partner: 106 (85.5) Child: 0 (0.0) Sibling: 3 (2.4) Parent: 3 (2.4) Friend: 12 (9.7)	Breast Breast	I: 18 (42.9) II: 12 (28.6) III: 4 (9.5) I: 46 (37.1) II: 40 (32.3) III: 12 (9.7)	5.8 years (3.9) 7.3 years (3.6)	L: 41 (95.3) M: 0 (0.0) M + (Re): 2 (4.7) R: 32 (74.4) H: 31 (72.1) L: 105 (84.7) M: 10 (8.1) M + (Re): 9 (7.3) R: 80 (64.5) H: 88 (71.0)
Chien et al. (2018), Taiwan	Longitudinal (T4 = 24 weeks)	T4 =46	*M* = 62.0 (7.8)	Female: 48 (100.0) Male: 0 (0.0)	Partner: 48 (100.0)	Prostate	II: 30 (62.5) III: 18(37.5)	Recruited when first diagnosed	S: 37 (77.1) R: 11 (22.9)
Cohee et al. (2017), USA	Cross-sectional	222	*M* = 47.98 (7.2)	Not reported	Partner: 222 (100.0)	Breast	Not reported	5.83 years (1.51)	Not reported
Dempster et al. (2011), UK	Cross-sectional	382	*M =* 62 (10.91)	Female: 257 (67) Male: 125 (33)	Partner: 359 (94.0) Other family: 23 (6.0)	Esophageal	Not reported	*Mdn* = 46 months (19–81)	Not reported
Graham et al. (2016), UK	Longitudinal (T2 = 12 months)	171	*M* = 62.56 (10.05)	Female: 124 (72.5) Male: 47 (27.5)	Partner: 165 (96.49) Other: 6 (3.51)	Esophageal	Not reported	*M* = 4 years (2–7)	Not reported
Hodges et al. (2009), UK	Longitudinal (T2 = 6 months)	101	*M* = 56.26 (30 – 76)	Female: 73 (72.3) Male: 28 (27.7)	Partner: 86 (85.1) Non-partner: 15 (14.9)	Head and neck	1-2 = 27 (60) 3-4 = 16 (35.6) 5 = 2 (4.4)	Not reported	Not reported
Janz et al. (2016), USA	Longitudinal^c^	510	<50 N = 70 (13.7%) 50-65 N = 218 (42.75%) >65 N = 222 (43.5%)	Not reported	Partner: 510 (100.0)	Breast	0 :125 (24.5) I-II: 388 (66.3) III: 46 (9.0)	4 years	L: 324 (63.6) (U)M:128 (25.1) (B) M:51 (10.0) R: 363 (71.1) C: 229 (44.9)
Kim et al. (2012), USA	Cross-sectional	455	*M* = 56.19 (13.01)	Female: 288 (63.3) Male: 167 (36.7)	Spouse: 305 (67.1) Offspring: 84 (18.5) Other: 66 (14.4)	Mixed^d^	Localized: 292 (64.2) Regional*:* 124 (27.3) Distant: 39 (8.6)	2.2 years (0.40)	Not reported
Maguire et al. (2017), Ireland	Cross-sectional	180	*M* = 57.3 (12.48)	Female: 136 (76.0) Male: 44 (24.0)	Spouse: 132 (73.4) Offspring or parent: 34 (18.8) Other:14 (7.8)	Head and neck	I-II: 81 (54.4) III-IV: 68 (45.6)	4.9 years (3.79)	S: 31 (17.2) C: 47 (26.1) R: 122 (67.4)
Mellon and Northouse (2001), Mellon et al. (2006, 2007), USA	Cross-sectional	123	*M* = 55 (14.5) (21-80)	Female: 80 (65) Male:43 (35)	Spouse: 65 (52.8) Child: 36 (29.3) Sibling: 10 (8.1) Significant other: 12 (9.8)	Mixed^e^	Not reported	3.39 years (1.0)	S: 108 (87.8) R: 48 (39.0) C: 28 (22.8) H: 4 (3)
Perndorfer et al. (2019), USA	Longitudinal (21-day diary)	69	*M* = 58 (10)	Not reported	Partner: 69 (100.0)	Breast	0: 8 (12) IA: 37 (53) IIA: 17 (25) IIB: 6 (9) IIIA: 1 (1)	5 months (2.09) after treatment	C: 21 (30) R: 50 (72) H: 58 (84)
Soriano et al. (2018a), USA Study (1)	Cross-sectional	46	*M* = 54.57 (13.31)	Not reported	Partner: 46 (100.0)	Breast	0: 11 (24) I: 17 (37) II: 15 (32) IIIa: 3 (7)	7.70 months after treatment	C and/or H: 15 (33)
Soriano et al. (2018a), USA Study (2)	Longitudinal (21-day diary)	72	*M* = 59.49 (10.34)	Male:70 (97) Female: 2 (3)	Partner: 72 (100.0)	Breast	0: 10 (14) I: 34 (47) II: 27 (37) IIIa: 17 (23)	5.77 weeks after treatment	C: 24 (33) H: 58 (81)
Soriano et al. (2019), USA	Longitudinal (21-day diary)	57	*M* = 60 (10)	Male: 55 (96) Female: 2 (4)	Partner: 57 (100.0)	Breast	0: 7 (12) IA: 30 (53) IIA: 14 (25) IIB:5 (9) IIIA: 1 (1)	12.2 months (1.9)	C:17 (30) R: 41 (72) H: 48 (84)
van de Wal et al. (2017), The Netherlands	Cross-sectional	168	*Mdn* = 67.4 (40-86)	Not reported	Partner: 168 (100.0)	Prostate	Not reported	*Mdn* = 7.5 years (0.9-20.0)	S:126 (75) S + R: 41 (25)
Wu et al. (2019), USA	Longitudinal (T1 = 6 months; T2 = 12 months)	62	*M* = 64.3 (8.4)	Not reported	Partner: 62 (100.0)	Prostate	Not reported	89.8 days (95.0)	R: 36 (52.2) S: 18 (26.1) B: 7 (10.1) R + B: 3 (4.3) S + R: 1 (1.4) WW: 1 (1.4) Missing: 3 (4.3)
Xu et al. (2019), China	Longitudinal (10 days)	54	Not reported	Not reported	Partner: 54 (100.0)	Breast	I: 22 (40.7) II:14 (25.9) III:18 (33.3)	22.1 months (19.88)	Not reported

a *Cancer survivors who identify as heterosexual women (HSW)*.

b *Cancer survivors who identify as sexual minority women (SMW)*.

c *Partners surveyed at Time 2 only*.

d *Mixed cohort = breast, prostate, colorectal, lung, ovarian, kidney, uterine, bladder, non-Hodgkin's lymphoma, skin melanoma*.

e *Mixed cohort = Breast, prostate, colon-rectal, uterine*.

*Treatment modality: S, Surgery; C, Chemotherapy; R, Radiotherapy; L, Lumpectomy; M, Mastectomy; M + Re, Mastectomy and Reconstruction; (U)M, Unilateral Mastectomy; (B)M, Bilateral Mastectomy; H, Hormonal therapy; B, Brachytherapy; WW, Watchful waiting*.

Out of the 16 studies, nine focused on partners (Janz et al., 2016; Cohee et al., 2017; van de Wal et al., 2017; Chien et al., 2018; Soriano et al., 2018a,b, 2019; Perndorfer et al., 2019; Wu et al., 2019; Xu et al., 2019), whilst seven studies reported data on caregivers, including other family members and friends. Caregivers were predominantly White, female, and middle-aged. Education level varied across studies, with college or above tending to be the most reported education level. Relationship length varied, with the longest mean length of relationship between caregiver and survivor being 43.0 years (range 8–57 years) (van de Wal et al., 2017) and the shortest being 24.40 years (*SD* 13.8) (Soriano et al., 2018a).

### Results of Assessment of Risk of Bias

The results of the assessment of risk of bias are outlined in Table 3, and indicate that most domains, including unbiased selection of cohort, validated measures of outcome and dependent variables, and appropriate analyses rated highly. Several limitations were identified in relation to study design, assessment of FCR and justification of sample sizes. Only five studies reported a sample size calculation (Mellon et al., 2006; Hodges et al., 2009; Kim et al., 2012; Maguire et al., 2017; van de Wal et al., 2017). Out of the 16 studies, only four studies followed caregivers up for an adequate period (defined as at least 12 weeks). However, half of the studies included for review were cross-sectional therefore could not be assessed against this criterion. Most studies provided adequate descriptions of the study cohort, but three studies provide limited demographic data (Dempster et al., 2011; Graham et al., 2016; Xu et al., 2019). Most used validated methods for assessing predictor variables; however, in one study (Soriano et al., 2018b), it was not clear if the measures used had been validated. Most studies used validated measures of assessing FCR; however, in four studies (Kim et al., 2012; Janz et al., 2016; Wu et al., 2019; Xu et al., 2019), it was unclear if adapted questionnaires had been validated. In one study, it was unclear if confounding demographic variables were controlled for in analyses (Chien et al., 2018).

**Table 3:** Assessment of risk of bias.

**Author**	**Unbiased selection of cohort?**	**Sample size calculation?**	**Adequate description of cohort?**	**Validated method for assessing predictor/ outcome variables?**	**Validated method for assessing fear of cancer recurrence?**	**Adequate follow-up period?**	**Missing data minimal?**	**Confounders controlled for?**	**Appropriate analyses?**
Boehmer et al. (2016)	●	○	●	●	●	N/a	●	●	●
Chien et al. (2018)	●	○	●	●	●	●	●	◐	●
Cohee et al. (2017)	●	○	●	●	●	N/a	●	●	●
Dempster et al. (2011)	◐	○	◐	●	●	N/a	◐	●	●
Graham et al. (2016)	●	○	◐	●	●	●	○	●	●
Hodges et al. (2009)	●	●	●	●	●	●	●	●	●
Janz et al. (2016)	●	○	●	●	◐	N/a	●	●	●
Kim et al. (2012)	●	●	●	●	◐	N/a	●	●	●
Maguire et al. (2017)	●	●	●	●	●	N/a	●	●	●
Mellon and Northouse (2001)	●	○	●	●	●	N/a	●	●	●
Mellon et al. (2006)	●	●	●	●	●	N/a	●	●	●
Mellon et al. (2007)	●	○	●	●	●	N/a	●	●	●
Perndorfer et al. (2019)	●	○	●	●	●	○	●	●	●
Soriano et al. (2018a) Study (1)	●	○	●	●	●	N/a	●	●	●
Soriano et al. (2018a) Study (2)	●	○	●	●	●	●	●	●	●
Soriano et al. (2019)	●	○	●	◐	●	○	●	●	●
Soriano et al. (2019)	●	○	●	●	●	○	●		●
van de Wal et al. (2017)	●	●	●	●	●	N/a	●	●	●
Wu et al. (2019)	●	○	●	●	◐	●	●	●	●
Xu et al. (2019)	●	○	○	●	◐	○	◐	●	●

*● = Yes; ◐ = Unclear, Partially; ○ = No; N/a = Not applicable*.

### Demographic Factors

There were significant associations between age and FCR. Twelve studies, reported in 13 articles, examined the relationship between age and FCR. Of these, one study found a weak negative association between age and FCR (*r* = −0.17) (Kim et al., 2012) whilst five studies reported a significant association which remained significant when other clinical and demographic variables were controlled (Mellon et al., 2007; Dempster et al., 2011; Maguire et al., 2017; van de Wal et al., 2017; Soriano et al., 2018a). However, no details regarding cancer stage and treatment type were reported in one study (Dempster et al., 2011). Four studies assessed the relationship between gender and FCR, one of which found a significant weak association between gender and FCR, with female carers reporting higher FCR than male carers (Maguire et al., 2017). Of the three studies that assessed the relationship between ethnicity and FCR, only one found a significant relationship, reporting that Latino partners were significantly more likely to worry than White partners, whilst Black partners were less likely to report worry (Janz et al., 2016). However, as this study used an adapted FCR measure, it is not clear if this has been validated. Seven studies assessed the relationship between education and FCR. Of these, one study found a very weak negative association between education and FCR (*r* = −0.16) (Cohee et al., 2017), however as there is no evidence of a sample size calculation, it is unclear if the study is sufficiently powered.

### Clinical Factors

#### Treatment

There was limited support for significant associations between time since diagnosis and FCR. Two out of the eight studies that assessed the relationship between time since diagnosis and FCR found that those caring for more recently diagnosed survivors reported higher FCR (Boehmer et al., 2016; Maguire et al., 2017) which remained significant when controlling for other demographic and clinical factors (Maguire et al., 2017). Of these studies, one study met all of the quality assessment criteria (Maguire et al., 2017), however sample size calculation was not reported in Boehmer et al. (2016)'s study, which may indicate issues regarding statistical power and potential for Type I errors.

Data demonstrated mixed support for significant associations between type of medical treatment and FCR. Seven studies assessed the relationship between type of treatment and FCR. Of these, one study reported a very weak positive association between chemotherapy and FCR (*r* = 0.14) (Maguire et al., 2017), whilst three studies reported significant results which remained significant after controlling for other demographic and clinical variables (Boehmer et al., 2016; Janz et al., 2016; Maguire et al., 2017; Wu et al., 2019). Those caring for survivors who had received anti-estrogen therapy (Boehmer et al., 2016) or chemotherapy (Janz et al., 2016; Maguire et al., 2017) reported higher FCR. Two studies found that those caring for survivors who had undergone major surgery were more likely to have lower FCR (Maguire et al., 2017; Wu et al., 2019). This finding was significant when controlling for other demographic and clinical variables at 6 months post-treatment, but not at 12-months (Wu et al., 2019).

#### Cancer Stage

Seven studies explored the relationship between cancer stage and FCR, none of which found a significant association (Mellon et al., 2007; Hodges et al., 2009; Kim et al., 2012; Boehmer et al., 2016; Janz et al., 2016; Maguire et al., 2017; Chien et al., 2018). One study assessed the relationship between cancer severity and FCR, and found a significant positive association when controlling for other demographic and clinical variables (Kim et al., 2012). However, as this study used an adapted FCR measure, its psychometric properties are unknown.

#### Comorbidities

There was limited support for associations between comorbidities and FCR. Of the five studies that assessed the relationship between comorbidities and FCR, two found that greater number of comorbidities resulted in higher FCR when controlling for other variables, specifically survivor comorbidities (Boehmer et al., 2016) and caregivers' own reported number of comorbidities (Janz et al., 2016). One study examined the relationship between FCR and survivor's physical health and found that increased caregiver FCR was associated with poorer physical health of survivors (Kim et al., 2012).

#### Medical Follow-Up

One study, reported in two articles, used a three week diary to investigate the impact of a mammogram on FCR, which reported that there was a significant increase in FCR during days leading up to the mammogram, and avoidance of threatening stimuli was predictive of FCR on the day of the mammogram (Soriano et al., 2019). However, it was not stated whether confounding demographic variables were controlled for in this analysis. Following the mammogram, partner responsiveness (response perceived as genuine and enthusiastic) predicted lower caregiver FCR, whilst patient capitalization attempts (disclosure of positive events) predicted greater FCR at week 3 (Soriano et al., 2018b). However, Soriano et al. (2018b) did not report a sample size calculation, therefore findings may be at risk of Type I errors.

### Psychosocial Factors

#### Emotional Distress

There were significant associations found between level of anxiety and FCR. Three studies assessed the relationship between anxiety and FCR, all of which reported a weak positive association between anxiety and higher FCR (*r* =0.24 to 0.39) (Kim et al., 2012; Soriano et al., 2018a, 2019)^2^. One study used the Hospital Anxiety and Depression Scale to examine the relationship between emotional distress (anxiety and depression combined) and FCR and reported a strong positive association (*r* = 0.73) (Hodges et al., 2009).

#### Interpersonal Factors

Data indicated mixed support for significant associations between survivors' and family caregivers' FCR (Table 4). Nine studies, reported in ten articles, assessed the relationship between survivors' and caregiver FCR. Of these, eight studies found weak to moderate associations between survivor and family caregiver FCR scores (*r* = 0.19 to 0.53) (Mellon and Northouse, 2001; Mellon et al., 2007; Kim et al., 2012; Boehmer et al., 2016; van de Wal et al., 2017; Soriano et al., 2018a, 2019; Perndorfer et al., 2019), which remained significant when controlling for other variables at 6 months post-diagnosis (Hodges et al., 2009). However, the quality of studies that report these findings are mixed, as six of the nine studies do not report a sample size calculation (Mellon and Northouse, 2001; Mellon et al., 2007; Boehmer et al., 2016; Soriano et al., 2018a, 2019; Perndorfer et al., 2019), whereas three studies did state this calculation (Hodges et al., 2009; Kim et al., 2012; van de Wal et al., 2017). Consequently, it is unclear if the aforementioned studies are sufficiently powered and are potentially at risk of Type I errors.

**Table 4:** Main study findings.

	**Dependent variable**		**Independent variables**		**Significant findings**
**Author, (year)**	**FCR score (SD)**	**Analysis**	**Non-psychosocial (demographic, clinical)**	**Psychosocial**	
Boehmer et al. (2016)	FRQ^a^ HSW^b^: 75.2 (13.6) SMW^c^: 71.8 (16.5)	Multivariate logistic regression	Sexual orientation; Co-residence; Years since diagnosis; Treatment type; Survivor comorbidities; Chemotherapy; Co-residence	Caregiver use of counseling in relation to cancer diagnosis; Social support (Survivor, Caregiver); Experience of discrimination (Caregiver); FCR score (Survivor)	*Non-psychosocial* Years since diagnosis (β = −0.25^***^); Anti-estrogen therapy (β = 0.22^***^); Survivor comorbidities (β = 0.25^***^) *Psychosocial* Caregiver and Survivor FCR: *r*_2_ = 0.29^***^ Social support (Caregiver) (β = −0.24^***^)
Chien et al. (2018)	MAX-PC^d^ 5.22 (1.78)	Multivariate logistic regression	Age (Patient); Religion (Patient, Partner); Employment status (Patient, Partner); Education level (Patient, Partner); Self-perceived health status (Patient); Treatment type (radiotherapy); Cancer stage; Living arrangement (Partner)	FCR score (Patient); Relationship satisfaction (Patient, Partner)	*Non-psychosocial* None. *Psychosocial* None.
Cohee et al. (2017)	CARS^e^ 11.794 (4–24)	Correlation; Mediation	Age (Survivors, Partners); Ethnicity; Education; Religion; Comorbidities; Time since diagnosis	Social constraints; Cognitive processing	*Non-psychosocial* Education *r* = −0.164* *Psychosocial* X (social constraints); M (cognitive processing) Indirect effect = 0.184, 95% bootstrap CI = 0.119 to 0.271. Direct effect = 0.038, *p* = 0.469, 95% CI = −0.066 to−0.142. [*F*_(3, 215)_ = 27.917, *R*^2^ = 0.280, *p* < 0.001]
Dempster et al. (2011)	CARS 13.93 (5.83)	Correlations; Regression	Age; Gender; Relationship to survivor; Months since diagnosis; Comorbidities	Anxiety; Depression; Illness perceptions: Acute/chronic timeline; Cyclical timeline; Treatment control; Emotional cause; Behavioral cause; Externalized cause; Consequences (Patient, Carer); Personal control (Patient, Carer); Illness coherence (Patient, Carer) Coping strategies (Reflection/relaxation, Positive focus, Diversion, Planning, Interpersonal)	*Non-psychosocial* Age (β = −0.171^***^) *Psychosocial* Cyclical timeline: *r* = 0.275^***^; Consequences (Patient): *r* = 0.306^***^; Consequences (Carer): *r* = 0.475^***^ Reflection/relaxation: *r* = 0.333^***^; Diversion: *r* = 0.327^***^; Interpersonal: *r* = 0.354^***^ Illness coherence (Carer) (β = −0.093*); Consequences (Carer) (β = 0.273^***^); Externalized cause (β = −0.124**) Reflection/relaxation (β = 0.165**); Positive focus (β = −0.107*); Interpersonal (β = 0.179**)
Graham et al. (2016)	CARS T1:13.65 (5.58) T2: 13.97 (5.59)	Hierarchical regression	Age; Gender; Relationship to survivor; Living arrangement; Months since diagnosis; Other illness/medical condition	Anxiety; Depression; IPQR-Cluster 2 vs. 1^f^; IPQR-Cluster 3 vs. 1; Coping strategies (Planning, Interpersonal, Relaxation, Positive focus)	*Non-psychosocial* None. *Psychosocial* IPQR-Cluster 3 vs. 1 (β = −0.205*); Interpersonal (β = 0.218*)
Hodges et al. (2009)	WOC^f^ T1: 11.77 (4.98) T2: 11.71 (5.21)	Correlations; Path analysis	Age (Patient, Carer); Gender (Patient, Carer); Relation to patient; Co-habiting status; Children; Employment status; Cancer site; Cancer stage	Anxiety; Depression; FCR score (Patient)	*Non-psychosocial* None. *Psychosocial* Carer FCR (3 and 6 months) *r* = 0.754^***^; Patient and carer FCR (6 months) *r* = 0.375^**^ Carer distress: *r* = 0.734^**^; (β = 0.20^**^); Patient FCR (3 months) (β = 0.18^**^); Carer FCR (3 months) (β = 0.69^*^)
Janz et al. (2016)	Worry scale^h^ *N* = 212 (47.1%)	Logistic regression	Age; Ethnicity; Education level; Health status; Comorbidities; Cancer stage; Treatment type (Chemotherapy, Radiation, Surgery)	Received enough information on risk of recurrence from health care providers; Emotional support from health care providers;	*Non-psychosocial* Non-Hispanic Black (β = 0.053^**^); Latino (higher acculturation) (β = 3.05^**^); Latino (lower acculturation) (β = 2.96^**^); One or more comorbidities (β = 1.95^*^); Chemotherapy (β = 2.77^**^) *Psychosocial* None.
Kim et al. (2012)	Adapted item^i^ −0.04 (0.99)	Correlations; Modeling analysis	Age (Survivor, Caregiver); Cancer severity	Anxiety; Quality of life (QoL): mental health and physical health (Survivor, Caregiver); FCR score (Survivor)	*Non-psychosocial* Age: *r* = −0.174^***^; Cancer severity (β = 0.197^***^) *Psychosocial* QoL Mental health (Caregiver): *r* = −0.296^***^; Anxiety: *r* = 0.239^***^; Survivor and Caregiver FCR: *r* = 0.19^***^; QoL Physical health (Survivor): (β−0.127^**^); Mental health (Caregiver): (β = −0.147^***^)
Maguire et al. (2017)	WOC 9.6 (5.82)	Correlations; Multiple regression	Age (Survivor, Caregiver); Gender (Caregiver); Time since diagnosis; Cancer stage; Treatment type (Surgery, Chemotherapy, Radiotherapy); Relationship to survivor; Employment status	Financial stress of caring; Time caring; Social support; Loneliness; QoL (Survivor)	*Non-psychosocial* Time since diagnosis: *r* = −0.18^*^; Chemotherapy: *r* = 0.14^*^; Extent of surgery: *r* = −0.25^***^ Age (Survivor) (*b* = −0.22^*^); Age (Caregiver) (*b* = 0.22^*^); Caregiver gender: (*r* = 0.21^*^); (*b* = 0.25^***^); Extent of surgery (*b* = −0.23^***^) *Psychosocial* Survivor QoL: *r* = −0.28^***^; Time caring: *r* = 0.34^***^; Loneliness: *r* = 0.27^***^ Financial stress of caring: (*b* = 0.20^*^); Time caring: (*b* = 0.37^***^); Loneliness: (*b* = 0.25^***^)
Mellon et al. (2006)	FRQ 73.1 (14.1)	Correlations	None.	Family stressors; Family hardiness; Social support; Family meaning of illness; Family QoL; Somatic concerns (Patient)	*Non-psychosocial* None. *Psychosocial* Family stressors: *r* = 0.29^*^; Meaning of illness: *r* = −0.28^**^; QoL: *r* = −0.29^*^
Mellon et al. (2007)	FRQ NR	Correlations; Modeling analysis	Age (Survivor, Caregiver); Gender (Survivor, Caregiver); Ethnicity (Survivor, Caregiver); Education level (Survivor, Caregiver); Role of relationship to survivor; Time since diagnosis; Other health problems	Concurrent family stressors (Actor effect, Partner effect); Family hardiness; Social support; Family meaning of cancer illness (Actor effect, Partner effect); Somatic concerns; FCR score (survivor)	*Non-psychosocial* Age (Partner effect): (β = −0.52^*^) *Psychosocial* Concurrent family stressors: *r* = 0.29^**^; Meaning of cancer illness: *r* = −0.28^**^; Survivor FCR: *r* = 0.41^***^ Concurrent family stressors (Actor effect): (β = 0.34^***^); Family meaning of cancer illness (Actor effect): (β = −1.24^**^); Survivor vs. family caregiver: (β = −4.89^***^)
Mellon and Northouse (2001)	FRQ NR	Correlations	None.	Family QoL; Family stressors; Family hardiness; Family social support; Family meaning of illness; Somatic concerns; FCR score (Patient)	*Non-psychosocial* None. *Psychosocial* Family QoL: *r* = −0.33^***^; Family stressors: *r* = 0.24^**^; Patient FCR: *r* = 0.40^***^; Family meaning of illness: *r* = −0.27^**^
Perndorfer et al. (2019)	FCRI^j^ Spouse evening FCR*:*1.43	Correlations	None.	Daily protective buffering (Patient, Spouse); Intimacy; Evening FCR score (Patient, Partner)	*Non-psychosocial* None. *Psychosocial* Protective buffering (Patient): *r* = 0.15^***^; Protective buffering (Spouse): *r* = 0.25^***^; Evening intimacy (Patient): *r* = −0.12^***^; Evening FCR score (Patient): *r* = 0.21^***^
Soriano et al. (2018a) Study (1)	Global FCR: CARS = 3.18	Correlations; Modeling analysis	Age (Patient); Patient physical symptoms	Social Constraints (Patient, Spouse); Anxiety; Depression; Relationship quality (Patient, Spouse)	*Non-psychosocial* Age (Patient) (β = −0.028^*^) *Psychosocial* FCR (Patient and Spouse): *r* = 0.53^***^; Anxiety *r* = 0.31^*^; Social constraints (Spouse): (β = 0.561^*^); Relationship quality (Spouse): (β = 0.050^*^)
Soriano et al. (2018a) Study (2)	FCRI = 1.51	Correlations; Modeling analysis	None	Social Constraints (Patient, Spouse); Negative affect (Patient, Spouse); Relationship quality (Patient, Spouse); FCR score (Spouse same day)	*Non-psychosocial* None. *Psychosocial* FCR score (Patient and Spouse): *r* = 0.22^***^; Social constraints: *r* = 0.27^**^; Negative affect: *r* = 0.32^**^ (DV: Same day FCR): Social constraints (Spouse): (β = 0.978^***^)^k^; Social constraints (Patient): (β = 1.088^*^); Negative affect (Spouse): (β = 0.496^**^) (DV: Next day FCR): Negative affect (Spouse): (β = 0.255^**^); Relationship quality (Spouse): (β = 0.091^*^)
Soriano et al. (2019)	FCRI Baseline^l^ = 5 (4). T1 = 1.117 (1.754) T2 = 0.840 (1.296) T3 = 0.570 (1.483)	Modeling analysis	None	Capitalization attempt (Spouse, Patient); Perceived partner responsiveness (Spouse); Event positivity	*Non-psychosocial* None. *Psychosocial* T3: Capitalization attempt (Spouse)^m^ (β = 0.488^**^); Patient capitalization attempt (Patient)^23^ (β = −0.662^**^); Perceived partner responsiveness^n^ (β = −0.421^**^)
Soriano et al. (2019)	FCRI 0.96 (1.78)	Correlations; Modeling analysis	None	Threat sensitivity (Patient, Spouse); Anxiety (Patient, Spouse); FCR score (Patient)	*Non-psychosocial* None. *Psychosocial* Patient FCR: *r* = 0.29^*^; Anxiety (Spouse): *r* = 0.39^*^; Threat sensitivity (Spouse): (β = 0.408^**^)
van de Wal et al. (2017)	CWS^o^ 12.6 (3.5)	Regression; Mean comparison	Age (Partner); Years a couple; Cancer history (Partner); Education level (Partner); Children; Time since diagnosis; Type of treatment	FCR score (Survivor); Health-related QoL (physical, social, physical role and emotional role functioning; mental health; vitality; pain; general health)	*Non-psychosocial* Age: (β = −0.295^*^) *Psychosocial* Survivor FCR score: (*r* = 0.44^***^); (β = 0.304^***^) High partner FCR vs low partner FCR: Emotional role functioning (*p* = 0.023^*^); Mental health (*p* < 0.001^***^); Vitality (*p* = 0.038^*^); General health (*p* = 0.042^*^)
Wu et al. (2019)	Cancer specific worry measure^p^ NR	Modeling analysis	Type of treatment (Radiation, Surgery)	FCR scores at baseline and six-months (Patient, Spouse)	*Non-psychosocial* Six-month time point: Surgery (β = −0.25^**^) *Psychosocial* Six-month time point: Baseline FCR (Spouse) (β = 0.62^***^) Twelve-month time point: Six-month FCR (Spouse) (β = 0.73^***^)
Xu et al. (2019)	Adapted measure^q^ 19.82 (17.77)	Modeling analysis; Mediation analysis	None	Illness representation; Daily Couple Communication (perceptions of positive and negative information)	*Non-psychosocial* None. *Psychosocial* Spouses' perception of positive information: (β = −0.168^***^); Spouses' perceptions of negative information: (β = 1.045^***^)

a *FRQ = Fear of Cancer Recurrence Questionnaire (Northouse, 1981). Higher scores indicate greater level of FCR (score range 22 – 110)*.

b *Caregivers of cancer survivors who identify as heterosexual women (HSW)*.

c *Caregivers of cancer survivors who identify as sexual minority women (SMW)*.

d *MAX-PC = Memorial Anxiety Scale for Prostate Cancer (Roth et al., 2003). Scale consists of 18 items, four-point Likert scale. Higher scores indicative of higher anxiety*.

e *CARS = Concerns About Recurrence Scale (Vickberg, 2003). Four items, ranging from 0 to 5. Higher scores indicative of greater FCR*.

f *IPQ Clusters: Cluster 1 = Carers have increasingly strong causal beliefs, particularly beliefs in emotional cause; Cluster 2 = Carers increasingly believe that they and the survivor understand condition, and feel over time that there will be less severe consequences for themselves and the survivor; Cluster 3 = Carers report decreasing belief in severe consequences for survivor and carer, increase in perception that condition is acute and increase in all control beliefs*.

g *WOC = Worry Of Cancer scale (Easterling and Leventhal, 1989). Total composite score from two items used ranged from 0-20*.

h *Adapted worry scale used in previous publications (Janz et al., 2011, 2014). Scores M = ≥ 3 considered “worriers”*.

i *Adapted from Zhao et al. (2009). measure. Higher score reflects greater FCR, zero score reflects moderate levels of FCR*.

j *FCRI = Fear of Cancer Recurrence Inventory (Simard and Savard, 2009). Six items ranging from 0-4. Higher scores indicative of greater FCR*.

k *Random effects greater but still significant*.

l *One week prior to diary period*.

m *N = 56 couples*.

n *N = 53 couples*.

o *CWS = Cancer Worry Scale, stipulating a cut-off score for high FCR as ≥14*.

p *Diefenbach et al. (1999). Mean of two responses calculated, higher scores indicated greater FCR*.

q *Five items adapted from prior research (Thewes et al., 2012), rated on a seven-point Likert scale*.

*Methods: Multivariate regression models analysis (regression, mixed models and generalized linear); Modeling analysis (path analysis, structure equation modeling and actor-partner interdependence model). NR = Not Reported*.

*** *p < 0.001*;

** *p < 0.01*;

* *p < 0.05*.

Two studies examined the association between relationship quality and FCR, with one study reporting a significant positive association which was also found in next-day FCR when measured over 21 days (Soriano et al., 2018a). Three studies, reported in five articles, assessed the relationship between social support and FCR. Of these, one study found that social support was significantly negatively associated with FCR when controlling for other variables (Boehmer et al., 2016). One study investigated the relationship between loneliness and FCR, reporting a weak positive association between loneliness and FCR (*r* = 0.27) (Maguire et al., 2017).

One study examined the relationship between negative affect (assessed using the Positive and Negative Affect Schedule) and FCR, which found that as spousal negative affect increased, so did FCR level (Soriano et al., 2018a).

Five studies investigated the impact of communication on FCR, all of which found significant results. Specifically, on a day that partners perceived the cancer survivor to be less available or responsive to discussions of cancer-related worries, partners were more likely to have greater FCR on that same day, but not the next day (Soriano et al., 2018a). One study found that patient disclosures of positive events resulted in decreased FCR as did partner responsiveness which was perceived to be genuine and enthusiastic (Soriano et al., 2018b). However, it is unclear if the adapted measure used to assess partner responsiveness is validated. Similar findings were reported whereby partners' perceptions of positive information (e.g., supportive and inclusive) and negative information (e.g., indifferent) resulted in a change in FCR (Xu et al., 2019). One study reported that cognitive processing mediated the relationship between social constraints and FCR (Cohee et al., 2017). Attempting to protect one's partner by hiding cancer-related concerns was weakly positively associated with increased FCR (*r* = 0.15) (Perndorfer et al., 2019).

#### Stress and Coping

Two studies, reported in four articles, assessed the relationship between stressors and FCR, all of which found significant results. Specifically, care-related stressors (financial impact and time-burden associated with caregiving) (Maguire et al., 2017) were positively associated with FCR, whilst a weak positive relationship was reported between stressors related to ill health and FCR (*r* = 0.24 to 0.29) (Mellon and Northouse, 2001; Mellon et al., 2006, 2007).

Two studies assessed the relationship between coping strategies and FCR. Of these, one study reported a weak positive association between interpersonal coping (e.g., seeking support from cancer survivor) and FCR (*r* = 0.35) (Dempster et al., 2011), whilst another study found that this association remained significant when other variables were controlled (Graham et al., 2016). Although the latter study indicated a 40% drop out rate over time, there were no significant differences on depression or FCR between participants who provided complete data and those who provided data at one time point only (Graham et al., 2016). One study found that increased use of reflection and relaxation was a significant predictor of higher FCR at 12 months follow-up, whilst those with a hopeful and in-control outlook exhibited lower FCR (Graham et al., 2016). The authors suggested that the association between increased use of diversionary and relaxation coping skills and greater anxiety may be indicative of such strategies reinforcing avoidance, which may be beneficial in the short term but maintains anxiety in the longer term.

#### Quality of Life

Four studies, reported in five articles, assessed the relationship between QoL and FCR. All studies found a significant result, indicating a weak positive association between QoL and FCR (*r* = −0.28 to 0.33) (Mellon and Northouse, 2001; Mellon et al., 2006; Kim et al., 2012; Maguire et al., 2017). Specifically, higher FCR was linked to lower QoL scores, including poorer caregiver mental health (Kim et al., 2012), lower survivor QoL (Maguire et al., 2017) and poorer family QoL (Mellon and Northouse, 2001; Mellon et al., 2006). One study found significant differences between health-related QoL in partners with high and low FCR, reporting that partners with high FCR obtained significantly lower scores on social functioning, emotional role functioning, mental health, vitality and general health (van de Wal et al., 2017). Most of the studies that reported on QoL met the key criteria of the quality assessment and reported on relatively large sample sizes ranging from 123 to 455.

#### Psychological Beliefs

One study, reported in three articles, examined the relationship between the meaning of illness and FCR, reporting a weak negative association between negative meaning of illness and FCR (*r* = −0.27 to −0.28; (Mellon and Northouse, 2001; Mellon et al., 2006, 2007). Three studies assessed the relationship between illness perceptions and FCR, all of which reported significant findings (Dempster et al., 2011; Graham et al., 2016; Xu et al., 2019). Specifically, one study found that an understanding of the disease was negatively associated with FCR, whilst belief of less serious consequences and control over condition were positively associated with FCR (Dempster et al., 2011). One study reported that caregivers with a reduction in beliefs of severe consequences and causes of the condition, and an increase in control beliefs and understanding of the condition was associated with decreased FCR over a 12-month time period (Graham et al., 2016). One study found that over a 10 days period, spouses' negative illness representations were negatively associated with their own disclosures of positive information (Xu et al., 2019). However, this study did not state a sample size calculation therefore statistical analysis may be underpowered and at risk of Type I error rates.

## Discussion

This review summarized cross-sectional and prospective quantitative research investigating the demographics, clinical and psychological factors associated with FCR in caregivers of cancer survivors. Sixteen studies, reported in 19 articles, were included and summarized narratively. Significant associations were found between FCR and certain non-modifiable factors, including younger age and treatment modality. Although there was only limited research investigating psychological processes (*n* = 3), significant associations were found between illness perceptions and FCR. Specifically, a good understanding of the cancer diagnosis was negatively associated with FCR, whilst belief of less serious consequences and control over the condition were positively associated with FCR.

There were mixed findings with regards to demographic factors and level of FCR. Younger age was significantly associated with FCR (Mellon et al., 2007; Dempster et al., 2011; Kim et al., 2012; Janz et al., 2016; Maguire et al., 2017; van de Wal et al., 2017; Soriano et al., 2018a), which may be due to the unexpectedness of cancer in younger age and the perceived negative physical, social or economic impact of such a disease (Llewellyn et al., 2008; Lebel et al., 2013). Limited significant outcomes were reported with regards to the remaining demographic factors. Similar findings have been reported in the cancer survivor literature, whereby no demographic, clinical or social factors reliably predicted subsequent distress in cancer survivors (Cook et al., 2018).

Of the 13 studies that assessed the association between clinical outcomes and FCR, six reported significant associations. Specifically, time since diagnosis (Boehmer et al., 2016; Maguire et al., 2017) was significantly associated with higher FCR, whilst which contrasts with the cancer survivorship literature (Crist and Grunfeld, 2013; Koch et al., 2013; Simard et al., 2013). Four studies found that treatment modality was significantly associated with FCR, which is consistent with the cancer patient and survivorship literature that indicates that different treatment approaches are significantly associated with FCR (Yang et al., 2017a,b; Maguire et al., 2018). Patients who have had chemotherapy or radiotherapy are likely to experience side effects, and an increased number of hospital trips and inpatient episodes, which may contribute to psychological morbidity (Denlinger and Barsevick, 2009). Furthermore, research has indicated that some patients may choose more invasive surgeries even when the risk of recurrence is low, in order to eliminate risk to the greatest possible extent (Williams and Jeanetta, 2016). Consequently, caregivers may perceive surgery as a more conclusive treatment, and therefore may be of the view that the cancer is less likely to return, as opposed to treatment side effects and multiple hospital trips which may act as triggers of FCR. Only one study explored the association between clinical follow-up (mammogram) and FCR, which reported a significant association (Soriano et al., 2019). As caregivers often attend medical appointments with the survivor (LeSeure and Chongkham-ang, 2015), it is likely that such follow-ups may also act as a trigger for FCR in caregivers.

Two studies reported a significant association between comorbidities and caregiver FCR (Boehmer et al., 2016; Janz et al., 2016). Internal physiological cues related to comorbid conditions may be misinterpreted as possible cancer recurrence, thus symptoms may act as a reminder of vulnerability and trigger FCR (Leventhal et al., 1980; Lee-Jones et al., 1997; Crist and Grunfeld, 2013). Caregivers are likely to witness survivors expressing somatic concerns and reporting treatment side effects, therefore, they may be more vigilant regarding changes in the survivors' physical health which may exacerbate worries that the cancer might return. Furthermore, lack of communication between the dyad may lead to worry regarding somatic concerns and side effects (Cohee et al., 2017; Soriano et al., 2018a). However, similarly to demographic factors, clinical indicators are not as critical as psychological factors in the development and maintenance of FCR, and there are intrapersonal factors which need to be considered.

Of the psychosocial factors examined, communication significantly affected FCR. The less someone was able to tell their partner about their cancer-related concerns, the more likely they were to experience FCR (Cohee et al., 2017; Soriano et al., 2018a). Unsupportive partner behaviors (i.e., critical or avoidant responses) are associated with both patient and partner reports of hiding concerns and disengagement (Manne et al., 2014). One study reported that caregivers hiding their own cancer-related worries in an attempt to protect the survivor was associated with increased FCR (Perndorfer et al., 2019). Caregivers can be reluctant to discuss emotions relating to cancer for fear of burdening or upsetting the patient (LeSeure and Chongkham-ang, 2015; Tolbert et al., 2018), but this may be contraindicated as a helpful strategy.

The review findings indicated that caregivers relied on various coping strategies, including reflection, relaxation, diversion and interpersonal approaches (e.g., through requiring frequent reassurance regarding FCR), which were significant predictors of higher FCR (Dempster et al., 2011; Graham et al., 2016). Research also highlights that caregivers engage in a high use of avoidance, distraction and denial (Papastavrou et al., 2012; Lambert et al., 2013), yet acknowledge that such strategies are only temporarily effective (LeSeure and Chongkham-ang, 2015). Consequently, it is likely that FCR is exacerbated and maintained as the psychological distress is not explicitly addressed.

Significant outcomes were reported for psychological processes, specifically illness perceptions (Dempster et al., 2011; Graham et al., 2016; Xu et al., 2019). Similar findings have been reported in the cancer survivorship, as illness perceptions have been associated with higher FCR and worry about cancer more generally (Corter et al., 2013; Park et al., 2013; Simard et al., 2013). Furthermore, individual interpretations or representations are often more influential than clinical characteristics in determining FCR (Llewellyn et al., 2008). However, a review of psychological distress in cancer survivors reported no consistent evidence that illness appraisals predicted longer-term distress (Cook et al., 2018).

This review provides preliminary evidence that theoretical models used to understand FCR in cancer survivors may also be applicable to caregivers. For example, the limited research investigating psychological beliefs indicates that illness perceptions explained additional variance in FCR when controlling for demographic and clinical characteristics. This provides support for the Common Sense Model (Leventhal et al., 1980) which states that individuals create cognitive and emotional interpretations of an illness threat, in order to appraise and determine if the threat is serious and requires attention. However, the limited explanatory power of the three studies that examined illness perceptions suggest that this model does not fully account for variance in FCR in caregivers of cancer survivors and indicate a need to look beyond illness perceptions. The broader blended model of FCR (Lebel et al., 2018) argues that triggers, perceived risk of recurrence and illness uncertainty predict FCR, whilst positive beliefs about worrying and intolerance of uncertainty act indirectly to increase FCR by increasing maladaptive coping. In this review, interpersonal factors such as communication and social support, as well as type of treatment and clinical follow-up were significantly associated with increased FCR, therefore lending support for the utility of this model in understanding FCR experienced by caregivers. Furthermore, the findings of this review suggest that caregivers implement maladaptive coping strategies such as diversion and reassurance seeking, which were significant predictors of FCR. Interventions that aim to reduce FCR in patients which focus on cognitive processing and metacognitions, rather than the content of thoughts, have been found to be more effective than traditional cognitive behavioral approaches (Tauber et al., 2019). Given that similar factors are reported to exist for caregivers as survivors, it may be that interventions based on the aforementioned theoretical frameworks may also be applicable to caregivers.

### Study Limitations and Implications for Research

There are several limitations which must be taken into consideration. As only published data were searched and included in this review, there is a possibility that relevant studies were missed. Furthermore, only citations written in English were considered for inclusion for review, which may have resulted in a language, selection or cultural bias. The aim of the review was to synthesize correlates and predictors of FCR in cancer survivors so as to produce a comprehensive overview of the current state of evidence with regards to factors associated with, and underpinning, FCR. As such, we only included quantitative studies. However, inclusion of qualitative studies may have provided valuable context or additional insights into the findings of this review. Meta-analysis of data was not possible to heterogeneity in included studies, which limited the depth of analysis possible. We focused, instead, on narratively summarizing the results of univariate and multivariate analyses, with preference given to the most complex/controlled analyses. However, this may make comparison with other literature difficult, and should be considered when interpreting findings.

Various methodological limitations of the included studies were identified. There is likely to be a risk of self-selection bias as recruitment methods were reliant on patients responding to the research adverts. Eight out of the 16 studies used a cross-sectional study design, thus precluding the ability to draw causal inferences. Only four of the prospective studies included in the review reported an adequate follow-up period; the remainder used experiential sampling methodology with follow-up periods ranging from 10 days to 3 weeks. Most studies reported data from the USA and participants were predominantly Caucasian females, thus may not reflect a representative sample of the population. It is also important to note that cancer patients were in different stages of diagnosis, therefore associations with FCR could differ as those caring for patients with more advanced cancer may perceive the diagnosis as being more serious and more likely to recur (Simard et al., 2013). Future research should attempt to address the observed limitations by recruiting larger, more representative samples of carers of patients with a range of different cancers.

With regards to the quality of studies, only five studies reported a sample size calculation, thus studies are potentially statistically underpowered and at risk of Type I error rates. Researchers should ensure that this is stipulated in future research papers, in order to ensure confidence in the statistical power of findings. Only three studies considered psychological beliefs associated with FCR in family caregivers (Dempster et al., 2011; Graham et al., 2016; Xu et al., 2019); further prospective research in this area is warranted.

### Clinical Implications

Health professionals may want to consider certain demographic and clinical factors, such as younger age and treatment modality, when offering information on treatment approaches and providing the space to discuss concerns about recurrence. Previous research has identified a need for planning for transition from patient to “survivor” (Gilbert et al., 2008; Houlihan, 2009), which involves discussions around treatment, ongoing management, managing FCR and identifying triggers for seeking help and support from healthcare team (Humphris and Ozakinci, 2006). Caregivers should be involved in care planning with the opportunity to discuss their fears about the cancer returning. Involvement in care planning would provide the caregiver with greater guidance on the most appropriate ways of supporting the cancer survivor. In cases where the patient does not want the caregiver to be involved in the care plan, caregivers should be offered their own support as the cancer experience can result in the caregiver adapting to a potentially altered future and sense of self (Tolbert et al., 2018).

## Conclusions

The results of the review indicate that caregiver FCR is a significant concern and highlights the importance of furthering current understanding of this prevalent issue. Weak to moderate associations were found between certain demographic and clinical factors and increased FCR. Further research examining modifiable factors are required, in order to enhance understanding of the psychological processes that are involved in the development and maintenance of FCR in caregivers of cancer survivors. By investigating modifiable factors, this will provide evidence and guide the development of appropriate and effective interventions for this population.

## Data Availability Statement

The original contributions presented in the study are included in the article/supplementary material, further inquiries can be directed to the corresponding author/s.

## Author Contributions

PF, MC, and LO'R conceived the study. MC and PF supervised the conduct of the review and provided extensive feedback on drafts. LO'R searched for data, screened and selected studies, extracted data, quality assessed included studies, and drafted the initial manuscript. AW screened and selected studies and cross-checked data extraction and quality assessment. SC provided clinical input into the review. All authors commented on a final draft of the manuscript.

## Conflict of Interest

The authors declare that the research was conducted in the absence of any commercial or financial relationships that could be construed as a potential conflict of interest.

## Footnotes

^1^The samples of 26, 27, 28, 38, and 39 were drawn from a larger database (SEER). Thirty-eight and 39 used the same sample and therefore will be considered as one study. Twenty-six−28 studied non-overlapping samples and therefore will be interpreted and referred to as separate studies. Similarly, samples of 29 and 30 were drawn from a larger study (OPA, UK) but will be interpreted as separate studies as they used non-overlapping samples. Thirty-one, 35, and 36 were based on data from the MDCSS database and used the same sample, therefore will be considered as one study.

^2^Anxiety was assessed using the Profile of Mood States – Short Form (34), the Hospital Anxiety and Depression Scale (27), and the PROMIS Anxiety Short Form questionnaire (38).
